# Data Mashups: Potential Contribution to Decision Support on Climate Change and Health

**DOI:** 10.3390/ijerph110201725

**Published:** 2014-02-04

**Authors:** Lora E. Fleming, Andy Haines, Brian Golding, Anthony Kessel, Anna Cichowska, Clive E. Sabel, Michael H. Depledge, Christophe Sarran, Nicholas J. Osborne, Ceri Whitmore, Nicola Cocksedge, Daniel Bloomfield

**Affiliations:** 1 European Centre for Environment and Human Health, University of Exeter Medical School, Truro, Cornwall TR1 3HD, UK; E-Mails: M.Depledge@exeter.ac.uk (M.H.D.); N.J.Osborne@exeter.ac.uk (N.J.O.); C.Whitmore@exeter.ac.uk (C.W.); N.Cocksedge@exeter.ac.uk (N.C.); D.Bloomfield@exeter.ac.uk (D.B.); 2 London School of Hygiene and Tropical Medicine, London WC1E 7HT, UK; E-Mail: Andy.Haines@lshtm.ac.uk; 3 Met Office, Exeter, Devon EX1 3PB, UK; E-Mails: brian.golding@metoffice.gov.uk (B.G.); christophe.sarran@metoffice.gov.uk (C.S.); 4 Public Health England, London SW1W 9SZ, UK; E-Mails: Anthony.Kessel@phe.gov.uk (A.K.); Anna.Cichowska@phe.gov.uk (A.C.); 5 School of Geographical Sciences, University of Bristol, Bristol BS8 1SS, UK; E-Mail: c.sabel@bristol.ac.uk

**Keywords:** data linkage, evidence base, environmental change, data platforms, climate change, surveillance systems, environmental health, ecological public health, big data, vulnerable populations

## Abstract

Linking environmental, socioeconomic and health datasets provides new insights into the potential associations between climate change and human health and wellbeing, and underpins the development of decision support tools that will promote resilience to climate change, and thus enable more effective adaptation. This paper outlines the challenges and opportunities presented by advances in data collection, storage, analysis, and access, particularly focusing on “data mashups”. These data mashups are integrations of different types and sources of data, frequently using open application programming interfaces and data sources, to produce enriched results that were not necessarily the original reason for assembling the raw source data. As an illustration of this potential, this paper describes a recently funded initiative to create such a facility in the UK for use in decision support around climate change and health, and provides examples of suitable sources of data and the purposes to which they can be directed, particularly for policy makers and public health decision makers.

## 1. Introduction

Climate change poses a range of threats to health and wellbeing on a global scale including: changes in the frequency and distributions of vector-borne diseases, increases in water and food-borne diseases, increases in malnutrition, and a range of health and wellbeing outcomes (including major short and long term mental health impacts) associated with extreme events [1,2,3,4,5]. Diverse strategies are needed to protect health, as far as possible, as climate change proceeds. These include strengthening both health surveillance and early warning systems for extreme events such as heat waves and floods. There is also an urgent need to explore causal pathways through which health consequences might arise.

Digital collection of data over the last 20 years has provided increasing database resources that have yet to be fully utilized to provide an evidence base for health outcomes and its linkage to environmental data. Leveraging from the increasing amounts of and access to these data, decision support tools have been used in business, health and the environment to support decision analysis and participation using interconnected databases and modeling capability around expected and unexpected events and impacts (e.g., http://www.espace-project.org/publications/Extension%20Outputs/EA/Espace%20Final_Guidance_Finalv5.pdf; https://www.gov.uk/government/publications/national-framework-for-nhs-continuing-healthcare-and-nhs-funded-nursing-care).

There is a growing evidence base (e.g., as summarized particularly, but not exclusively, by the World Health Organization (WHO) and the United Nations (UN) Intergovernmental Panel on Climate Change (IPCC)) on the potential impacts and adaptation strategies to reduce health impacts. There is guidance from the WHO on how to estimate health and adaptation costs [6; http://www.euro.who.int/en/health-topics/environment-and-health/Climate-change/publications].

Numerous data sources and decision support tools could be employed in the study of potential impacts of climate change and variability on health and to enhance the development of more effective adaptation strategies. In addition, there is rapidly increasing interest in the health “co-benefits” and the “co-harms” or negative unintended consequences of policies; there are also illustrative case studies around reducing greenhouse gas emissions (e.g., European Union funded projects URGENCHE (http://www.urgenche.eu) and PURGE (http://purge.lshtm.ac.uk)), reflecting evolving understanding of the cost effectiveness as well as other benefits of policies that tackle complex environmental, societal and human health challenges [7,8,9,10,11].

Earlier proposals for linking health and environmental data to illuminate the effects of global environmental change have highlighted the limitations of traditional epidemiological monitoring of disease and mortality for this purpose [12,13,14,15,16]. These included: the significant spatio-temporal lags between changes in climate and health outcomes; the potential for confounding bias associated with changes in socioeconomic factors and health care delivery; and the effects of spontaneous or planned adaptation. Five key challenges (see [12]) for developing monitoring systems for the health impacts of global environmental change have been outlined. These encompass: defining biological, environmental and human health indicators; assessing the data needed to monitor these indicators; ensuring the availability of technology for measuring them; involving organizations that can provide appropriate data and defining the gaps that could be addressed by new developments. In this paper, we give examples of approaches to addressing the first four of these challenges in the context of a new initiative to link health and environmental (particularly weather and climate) data in the UK. 

The potential resources required for decision support around climate change and human health range from: existing health, socioeconomic and environmental databases (including those with horizon scanning capability, and forecasting capabilities); resources for searching and weighing the evidence base; examples of vulnerable communities and subpopulations, and of case studies of adaptation and resiliency (both successes and failures) and other assessments; and networks of researchers/experts including those with the expertise to undertake evaluation of interventions. This will be supplemented by the ability to assess large scale interventions and natural experiments. Also, the long timelines needed to study both climate change and its subsequent health impacts suggest that access to archived historical data will be necessary to allow analysis of slower, more subtle changes in ecological and human health outcomes that might otherwise be missed, especially the growing global burden of chronic disease. In this case though, it will be important to take into account the potential for changes in diagnostic criteria over time and in the detection of health outcomes.

This complexity presents an increasing challenge to the researcher and policymaker in understanding and addressing the possible risks and benefits to human health and wellbeing from climate change, together with devising effective strategies to reduce risks and to promote more sustainable patterns of development. A further complicating factor is that the mechanisms through which climate change can affect health and wellbeing are varied, ranging from changes in the weather, to secondary effects such as the distributions of pathogens and pollutants, and the psychological effects associated with fear of climate-driven events such as severe storms and flooding. Nevertheless, new technology and system developments, particularly around “data mashups”, as well as growing interdisciplinary and inter-institutional research and resources, have increasing potential to produce the evidence base and decision support necessary to explore the interconnections, the intended and unintended consequences of interventions to increase resilience, and impacts of climate change and human health.

## 2. Specific Climate Change and Human Health Data Challenges

Climate change presents its own specific challenges in terms of the evidence base and decision support. The relevant data for the evidence base are increasingly derived from many different complex sources and data types (including climate, weather, environmental, and human health and wellbeing data). The impacts of climate change are seen in broad temporal and geographic scales which will likely affect a wide range of environment and health outcomes [1,2,13,14,15,16,17]. Furthermore, historically the climate, weather, and environmental research communities have not worked closely with the health and wellbeing research communities; this is changing with the increasingly obvious pressures of climate and other environmental changes, as well as through initiatives such as “ecological public health” and “ecosystems health”, that are bringing these different research communities together [18,19,20]. Nevertheless, training and funding for the necessarily interdisciplinary research required to explore this complex evidence base are still inadequate.

One major issue with all these types of data, is that the user is at the mercy of the types of historical data already collected. These previously collected data may not be appropriate to answer the questions of today, much less the future. This includes both the range and types of variables, and the granularity or resolution of temporal and spatial data. For example, historic pollen data may have been collected at relatively few sites and for only a few types of pollen over a large geographic area, not allowing for analyses to evaluate possible associations with geographically detailed asthma emergency room admission data or for health associations with specific types of pollen. 

The amounts of both environmental and health data are growing in size, detail and complexity. These data can now be processed, analysed, and stored in increasingly accessible formats thanks to major improvements in computing hardware and data management software. Nevertheless, keeping track of all these data is very challenging on several levels for current owners and potential users of particular databases, notably the challenges of keeping up with the ever increasing availability of new databases, their documentation, and potential resources (e.g., new analysis approaches). Furthermore, the potential to link various databases raises other important issues (such as confidentiality and access arrangements, particularly for health databases) which are discussed below.

Environmental data are often collected over much longer time scales and with greater frequency (e.g., daily or even hourly rainfall data or oceanographic data) over large geographic areas compared with the health and wellbeing data. The latter data are often collected over much shorter time periods and/or clustered at particular points in time in relatively small geographic areas (*i.e.,* commonly at a single time period entry to a prospective cohort study at baseline, discrete follow up interactions, or at a patient’s interaction with the health service). For example, the Avon Longitudinal Study of Parents and Children (ALSPAC) is a longitudinal study of 14,000 mothers living in the Avon Valley (UK) enrolled during pregnancy in 1991 and 1992 following the health and development of their children with periodic data collections until the present day; the US National Health and Nutrition Examination Survey [NHANES]) examines new representative samples of the entire US population on an approximately annual basis.

The types of data collected to evaluate health and environmental issues are also very different, ranging from measures of an individual’s wellbeing (e.g., mental health) to remote sensing data of an entire country’s coastline. Furthermore, both the logistical links (*i.e.,* which variables to actually link between health and environmental databases) and the potential causal associations (e.g., ocean currents and human diseases) between the different types of data still need to be worked out, except for a few cases (e.g., extreme temperatures and mortality) [21]. Finally, the impacts of climate change on human health are being felt first in developing areas around the world, where there are the least data and other resources available to monitor potential cause and effect relationships. 

### 2.1. Statistics and Analysis

Many statistical methods used by the environmental and human health research communities are different, although increasingly tools (such as geographic information systems (GIS)) and methodologies (such as Bayesian analyses) are seen in both research areas. Nevertheless, new and complex analyses and models are needed to fully explore the linkages between climate change and human health. For example, there is a need to be able to accurately predict near term extreme climatic events never previously experienced by humanity (e.g., the 2012 “Super Storm” Sandy and 2013 Typhoon Haiyan) with sufficient warning to permit effective countermeasures to be implemented; and to project potential health and environmental impacts under different future scenarios (e.g., the changing demands for air conditioning or other protection against thermal extremes and for pharmaceutical use with rapidly aging populations under conditions of climate change) [22]. There is also an ongoing need to understand the extent of uncertainty engendered by combining different types of data and modeling approaches.

Geographical Information Systems (GIS) provide substantial support for the management and availability of (spatial) data. GIS have undergone considerable changes over the past decade, with commercial GIS packages progressing from stand-alone software packages to the development of GIS applications for desktop, server, web, and mobile GIS, not to mention the inclusion of Cloud Computing. Similar trends have been observed in the development of Open Source GIS. As Evans and Sabel (2012) have demonstrated, extensive spatial analytical functionality can now be incorporated to web GIS [23]. For example, PostgreSQL (coupled with PostGIS) and MySQL are two popular open source database management systems (DBMS) widely used for GIS applications. These DBMS may be integrated with the MapServer (http://www.mapserver.org) and GeoServer (http://www.geoserver.org) packages to provide open source WebGIS, with limited functionality. Furthermore, data can be shared and exchanged through metadata harvesting, analysed directly on web platforms, and/or users can access download services to obtain digital copies of stored data.

The standardisation of spatial data services by the Open Geospatial Consortium (OGC) (http://www.opengeospatial.org) has enabled interoperability been systems, allowing for the sharing of spatial data amongst web mapping portals, as well as the consuming of data services by desktop GIS. Of note, recently the OGC has initiated a Health Domain Working Group in response to the increasing use of geospatial data in a range of health applications (http://www.opengeospatial.org/node/1823). The Web Processing Service (WPS) (http://www.opengeospatial.org/standards/wps) takes this a step further where the actual processing of spatial data can be published and shared across the web. Similar Metadata standards have been derived, allowing for the harvesting of data sets between data management systems, enhancing the capabilities of data discovery, and therefore the linkage of data.

Major multi-national corporations interested in the management of spatially enabled data, such as Google Inc., are now leveraging these developments via inter-linked databases and mapping products to provide tools to users over the web to be able to query and explore data. The plethora of environmental, health and social data, and the tools to analyse them now becoming available on the web, combined with both a growing familiarity with Web 2.0 and an increasing workforce of non-geographically trained experts in WebGIS, have led to a further development in the visualisation of these data over the web. The use of “mashups” of spatially enabled data from a variety of sources, provides the opportunity to use the additive power of datasets to infer results more revealing than the individual datasets allow.

## 3. Climate-Environment-Health Data Mashups

Improved data linkages between climate, weather, and environmental data with the health and wellbeing databases, and expanded access to these linked data offer a powerful decision support tool, often called a “data mashup”. A “mashup” (a term originally derived from jazz) implies easy and fast integration of different types and sources of data, frequently using open application programming interfaces and data sources, to produce enriched results that were not necessarily the original reason for assembling the raw source data [24]. Data mashups can lead to new and innovative uses of data by a wide range of individuals and institutions.

A data mashup of accessible and linked integrated health-environmental data applied to the human health and wellbeing impacts of climate change would provide both the evidence base and decision support tools through:

- Facilitating novel research into environmental exposures and health (including “natural experiments”) using integrated models to detect and attribute changes in health with changes in climate and other environmental variables;
- Rapidly identifying “hot spots” (locations and points in time with convergent increased environmental and human health risks to vulnerable populations;
- Providing healthcare practitioners, public health planners, and environmental managers with relevant surveillance and other information for improving services for locations and populations identified as being at risk;
- Initiating and evaluating interventions to promote adaptation (and unintended adverse consequences) by reducing the exposures, and thereby the health effects at both the individual and population levels;
- Disseminating and providing access to data as part of outreach and engagement with the research community, policymakers and civil society;
- Providing novel perspectives, allowing a greater understanding of the effect of climate change on human health within the context of ecosystem health;
- Fostering resilience and adaptive capacities for individuals, households, communities, and regions to the health and wellbeing impacts of climate change by scaling up adaptation strategies of proven effectiveness.

Ideally, these linked data should be available through a website portal developed to facilitate access, as well as dissemination and engagement, while preserving the confidentiality of the data (the latter a major issue for health and other types of data as described below). The portal can also serve as both a data repository and data analysis “space,” with ongoing user commentary and data and user documentation. The data mashup portal should ideally be able to be queried (“query-able”), and provide visualisation, mapping, and other functions for different types of users to explore and display data. Furthermore, if the data are collected and analyzed in real time, then many of the functions described above could be delivered for a range of stakeholders (including public health planners and policy makers) to make decisions in real time.

### 3.1. Examples of Existing Programmes with Focus on Linking up Different Types of Data

Historically, health registries (especially in Scandinavia), as well as health systems (including health insurance), and administrative databases for government, civilian industry, and the military, have had the capacity to explore and link large amounts of disparate data in time and space (http://www.kvalitetsregister.se/om_kvalitetsregister/quality_registries; http://rekisteritutkimusen.wordpress.com/registers/register-controllers/; http://www.swpho.nhs.uk/skincancerhub/about/default.aspx).

There are some existing examples of linking healthcare data to environmental data to facilitate the study of climate, weather, air pollution, and health relationships. One such example is the use of the General Practitioner (GP) Research Database in the UK to study associations between primary care consultations and environmental exposures (e.g., cold weather and GP consultations for respiratory diseases in the elderly; air pollution and daily GP consultations for allergic rhinitis; and for thunderstorm-related asthma) [25,26,27]. While these studies have laid a foundation of research in this area, they have primarily focused on understanding relationships between health and the environment that led to exacerbations of existing conditions; they have not tackled the public health goal of using data to reduce initiation of disease (e.g., by early preventive interventions), hence eliminating the risk of future exacerbations of disease that require the attention of the healthcare system. Furthermore, there have been relatively few efforts to foster and study resilience and adaptive capacities for individuals, households, communities, and regions to the health effects of climate change.

More recently, there are new examples of the joining up of academia, industry, and government to pursue large data linkage projects for a range of purposes (as detailed above). This has the advantage of researchers approaching questions from a perspective that stakeholders understand, and can seamlessly feed into the policy cycle, hence having a pathway to impact. However, particularly (but not exclusively) with health data, there are many inherent issues which need to be addressed before such linkages can be undertaken, the most important of these are to ensure that confidentiality of data for individuals, communities and subpopulations is protected while at the same allowing appropriate use. These issues are in turn related to who has access, and who has control of these data. Furthermore, there can be unintended uses of these data mashups, such as in the US where publicly available linked health data from the National Health Interview Survey (NHIS), used widely by researchers and for health policy, were used to inappropriately identify individual participants leading to subsequent highly restricted data access [28,29]. In addition, access to these shared resources is becoming more costly, in part due to the costs of processing and storage as well as to the general absence of continuous research funding (unless there is demonstrated commercial potential) [30].

In Table 1, we have listed some examples of sources of climate, weather, and environmental data and health and wellbeing data currently available in the UK and beyond. In general, these sources have not been joined up, but exist as stand-alone resources (especially with the divide between health *vs.* environmental), each with their own access arrangements and geographical scope, even though there may be geo-temporal overlap between these data potentially allowing linkage and mashups. Furthermore, several have time-limited research funding, leading to a lack of resource continuity. Several new projects/programmes have begun recently with the intent of either serving as a central source of information or metadata (e.g., new NOAA Metadata Access Tool for Climate Change and Health (MATCH) Programme http://match.globalchange.gov/geoportal/catalog/main/home.page), or even the repository of data. These include a few with an emphasis on the linkage of health and environmental data, and even fewer on future access to these linked data by researchers and other stakeholders (e.g., EXPOSOMICS Project http://scitechdaily.com/exposomics-looks-to-tie-environmental-exposure-to-biological-triggers-of-disease/; and see **Box 1** on the MED MI Project). 

**Table 1:** Exemplar database linkages/mashups of climatic and environmental with human health data.

Institution/Project	Brief Description and Links
EVO	The Environmental Virtual Observatory (EVO) is a proof of concept project with NERC funding that has been created to demonstrate that linking data, models and expert knowledge will provide cost effective answers to vital wide-ranging environmental issues, initially in the soil-water system. The project exploits cloud computing to develop new applications for accessing, filtering and synthesising data to develop new knowledge and evaluation tools. It investigates possible structures for the cloud environment and develops exemplars at a local and national scale to demonstrate how the EVO could make environmental monitoring and decision making more efficient, effective and transparent to the whole community. http://www.nerc.ac.uk/research/programmes/virtualobservatory/
ECDC E3 Geoportal	The objective of European Centres for Disease Control (ECDC) E3 Geoportal is to promote geospatial infectious disease modelling in Europe and its integration in public health. There are many different determinants of infectious disease transmission but they are often highly dispersed and/or difficult to obtain. The E3 Geoportal will facilitate the collection and exchange of these datasets in a user-friendly manner. It is an inventory of information and resources which are collected, maintained, and managed by a collaborative effort under the European Environment and Epidemiology Network. https://e3geoportal.ecdc.europa.eu/SitePages/Home.aspx
SAIL (Wales)	The Secure Anonymised Information Linkage (SAIL) Databank is a large scale data warehouse technology. The SAIL system links together the widest possible range of person-based data using robust anonymisation techniques on the College of Medicine’s IBM supercomputer and bespoke data transportation fabric to a wide range of NHS systems in Wales, allowing for future data mashups. SAIL is continually expanding, both in types of dataset and in geographical coverage, and many additional organisations have since provided, or agreed to provide, their datasets. Through the robust processes that have been developed and implemented, this growing databank represents a valuable resource for health-related research and service development, whilst complying with the requirements of data protection legislation and confidentiality guidelines. http://www.ehi2.swansea.ac.uk/en/sail-databank.htm
URGENCHE Project	Urban Reduction of GHG Emissions in China and Europe (URGENCHE) is a FP7 funded project bringing together a team of internationally recognised scientists to develop and apply a methodological framework for the assessment of the overall risks and benefits of alternative greenhouse gas (GHG) emission reduction policies for health and well-being in China and Europe. http://www.urgenche.eu
NOAA MATCH	NOAA Metadata Access Tool for Climate Change and Health (MATCH) is a publicly accessible, online tool for researchers that offers centralized access to metadata (standardized contextual information) about thousands of government-held datasets related to health, the environment, and climate-science. http://match.globalchange.gov/geoportal/catalog/main/home.page
PULSE-Brazil	NERC-funded project involving the University of Exeter, the Met Office and Brazilian partners. PULSE-Brazil brings together health data (especially respiratory health) and environmental data. It uses different kinds of data (e.g. satellite records on fires in the Amazon) and it has a different main output (a tool to support decision makers, rather than a platform to aid researchers). Both projects can learn from each other across a range of technical, methodological and theoretical issues. http://gtr.rcuk.ac.uk/project/E994D2D9-6A89-4F14-9C70-28076CCFBBBE
ESCAPE	EU funded project on long-term health risks to air pollution exposure. ESCAPE concentrates on respiratory, cardiovascular, cancer and pregnancy-related risks. The project’s communications strategy concentrates on producing material for use with patient groups. http://www.escapeproject.eu
AVOID	The Met Office Hadley Centre has datasets produced under the DECC/Defra funded Avoiding Danger Climate Change (AVOID) Programme. Includes observations programme to measure salinity, current velocity and temperature in the upper oceans. http://www.metoffice.gov.uk/avoid/
EO2HEAVEN	EO2HEAVEN (Earth Observation and Environmental Modelling for the Mitigation of Health Risks) was a research project co-funded by the European Commission as part of the 7th Framework Programme (FP7) Environmental theme. EO2HEAVEN contributed to a better understanding of the complex relationships between environmental changes and their impact on human health. The project monitored changes induced by human activities, with emphasis on atmospheric, river, lake and coastal marine pollution. The result of this collaboration was the design and development of a GIS-based system upon an open and standards-based Spatial Information Infrastructure (SII) envisaged as a helpful tool for research of human exposure and early detection of infections. http://www.eo2heaven.org
EXPOSOMICS/HELIX	EXPOSOMICS is an EU funded project, led by Imperial College, and involving institutions from six other countries. It aims to predict individual disease risk from examining drinking water and air-borne contaminants; health data (long-term cohorts) and environmental data will be analysed together. HELIX project is an EU funded project, led by the Centre for Research in Environmental Epidemiology (CREAL) involving institutions from eight other countries. It is focused on the early life exposome since pregnancy and the early years of life are well recognized to be periods of high susceptibility to environmental damage with lifetime consequences. http://www.projecthelix.eu/en/news/item/4-ec-fp7-exposome-programme-launch-at-who-iarc

**BOX 1. MED MI: Linking Human Health and Wellbeing with Weather, Climate, and the Environment**

As an example of the use of data mashups to provide the evidence base and decision support tools for climate and health, the UK Medical Research Council and the UK Natural Environment Research Council have funded a new strategic partnership, the MED MI Platform (**M**edical and **E**nvironmental **D**ata—a **M**ashup **I**nfrastructure) (http://www.ecehh.org/research-projects/medmi/) to create the unique central data and analysis facility with exceptional data resources and analysis skills capable of driving novel research into the relationships between climate, weather, environment, and human health and wellbeing; and to disseminate this shared resource to medical and public health researchers, in the UK and beyond. The strategic partnership of the Met Office, Public Health England (PHE), the London School of Hygiene and Tropical Medicine (LSHTM), and the University of Bristol led by the University of Exeter Medical School, has access to a range of climate, weather, environment, and human health and wellbeing data which will be linked through geo-spatial variables (see Table 3, Table 4 and Table 5). These data will be made available on a web-based platform, initially to the researchers as they perform a series of feasibility demonstration projects as well as “beta test” the platform and logistics (particularly data confidentiality); in the future, the MED MI Platform will be made available to other researchers, especially those interested in linking other environment and health databases to expand the MED MI database to explore different environmental change (including climate) and health issues. At the same time, the researchers will be interacting with a range of stakeholders (particularly researchers and policy makers) to explore different interfaces between the user and the data to expand the uses and usefulness. There is also an interdisciplinary and multi-institutional Advisory Board which can provide guidance on priorities. In addition, the MED MI Partnership will explore the rapidly growing number of similar potential national and international data mashups (Table 1) for additional linkage and collaboration opportunities as well as lessons learned and around analysis and governance best practice as well as which may lead onto new funding opportunities.

Many policies in sectors such as electricity generation, housing insulation and ventilation, urban transport, food and agriculture can lead to reductions in emissions and provide ancillary benefits for human health (*i.e.*, health co-benefits). Examples include: reduced fine particulate air pollution from decreases in coal combustion or low emission motor vehicles, increased physical activity as a result of increased active travel in urban areas, increased uptake of low emission (e.g., due to reduced consumption of ruminant meat) healthy diets. Potential co-harms could include increased indoor air pollution (e.g., environmental tobacco smoke, mold, house dust mites, or radon) from tightly sealed dwellings. Data from a range of sources could be used to monitor the transition to a low carbon economy and the resulting health co-benefits, however this article focuses on data related to climate change impacts on health [31,32,33,34,35].

### 3.2. Potential Future Uses of Linkages between and among Health, Environmental, and Climatic Data

As noted above, there is a large and growing body of work on climate, weather, and environmental data around climate change, while there is relatively little, often still speculative, work with regards to health outcomes. The health outcomes which are of particular interest in developed countries in the light of current knowledge are diverse and include: heat/cold related deaths/morbidity; a range and impact of climate-sensitive vector borne, food borne, and water borne communicable and non-communicable diseases (e.g., Lyme disease); mortality and mental health outcomes in association with extreme events (e.g., heat waves, floods, droughts, *etc.*); respiratory and cardiovascular disease events associated with air pollution (e.g., ozone); allergic diseases associated with pollen; a range of health outcomes possibly associated with algal biotoxins [1,36,37,38,39,40]. A major concern for low income countries is increased under-nutrition due to reduced crop production (particularly in tropical and sub-tropical regions) [41,42]. 

A priority is to identify which populations are most vulnerable or, conversely, most resilient to the effects of climate change on human health and other outcomes based on demographic, socioeconomic, environmental risk factors, and/or geographical characteristics. The ability to reliably identify these populations would indicate where to focus resources for health outcome surveillance purposes, as well as exploiting existing health and wellbeing databases including long term cohort studies. In addition to exploring known associations (such as urban heat deaths, particularly in the deprived elderly), the linkage of health and climate data could be used to define and identify new vulnerable populations or time periods of vulnerability for specific populations; for example, whether winter mortality in the preceding year influences heat-related mortality in the following summer, and if so, whether particularly susceptible populations can be identified [43]. As we are not especially well adapted to living in current climatic conditions (as is evident from the human tragedies caused by the numerous severe storms, floods, heat waves, *etc.*, that we regularly witness), there may already be data available to guide us in what to look for and measure in the future as well as identifying those populations that are better at adapting.

Furthermore, these data can be used to explore both the effectiveness and unintended consequences of public health interventions such as heat wave early warning systems in known and newly identified vulnerable and resilient populations. Such data could also be used to study the long term effects of climate-related extreme events such as floods. For example, in a study of floods in the UK, mortality data, geo-referenced by postcode of residence, were linked to a national database of flood events for 1994 to 2005 [44]. The ratio of mortality in the post-flood year to that in the pre-flood year within flooded postcodes was compared with that in non-flooded boundary areas (within 5 km of a flood). Counter-intuitively, a deficit of deaths was found in the year following flooding, perhaps because of population displacement caused by flooding; further work is needed to clarify this and many other questions.

The linked data could also be used to study trends in the incidence and geographic variation of various climate-sensitive infectious and vector-borne diseases (e.g., Lyme, salmonella, legionella, campylobacter) in relation to short and long term variability in weather (e.g., temperature and precipitation), as well as longer term changes in climate, taking into account the potential confounding factors (e.g., changes in patterns of physical activity which can change exposure to ticks transmitting Lyme disease) [45,46]. In addition to identifying and mapping changing trends in infectious diseases, potentially these analyses could identify new and important associations between specific climatic patterns and specific infectious diseases, as well as the development of predictive models looking at potential tipping points and triggers. Again the potential to do these analyses in real time may allow for active prevention of these infectious diseases in the future.

Another key area of growing interest is how climate change is affecting the distribution, bioavailability, fate, and persistence of anthropogenic pollutants in the environment, which in turn alter patterns of human exposure, routes of exposure, and toxic effects resulting in acute and chronic diseases [47]. There are extensive databases that capture current levels of contamination in soil, sediment, water, biota, and human tissue samples which may be useful in this regard. This serves again to illustrate the complex links and diverse mechanisms by which climate change results in effects on both human health and on ecosystem structure and function, as well as the potential value of the mashup approach in bringing such causal chains to light.

There has been relatively little work done on the issue of potential positive “co-benefits” or negative co-harms of climate change adaptation strategies for human health. For an example of a co-benefit of climate change, we know that currently those living closer to the coast enjoy better health and wellbeing than those inland perhaps due to greater levels of outdoor physical activity; increasingly warm weather may lead to more people spending more time out of doors exercising in the natural environment [48]. On the other hand, one example of a co-harm is when coastal adaptation sites become colonized by mosquitoes, leading to an increase in mosquito-borne diseases especially for people exposed in the natural environment through work or recreation [49].

Understanding how adaptation requirements are likely to change in the coming years as climate change progresses will be valuable with regards to developing adaptation strategies. In particular, health impact assessments of adaptation strategies can help to ensure that harms do not inadvertently occur; and in these specific cases, to monitor insect vectors and changes in levels of outdoor activity. 

Finally, the data linkages provided by environmental-health mashups could explore currently hypothetical but unproven associations between climate and health, such as climate change, harmful algal blooms, and human health effects [50]. Harmful algal blooms (HABs) and their potent natural toxins have been associated with a range of diseases, ranging from gastrointestinal illness and asthma exacerbations to an increased (although controversial) risk of neurodegenerative diseases (e.g., ALS, Alzheimer’s) [39]. HABs appear to be increasing in all aquatic ecosystems worldwide, associated with increased nutrients and possibly climate change. Many algal species produce blooms, only some produce toxins; in the context of climate change, these species are expected to mix, and their tendency to form blooms to change in the future [51]. In order to explore the associations between climate change, HABs and human health effects, there will need to be data linkages between coastal and oceanographic data (e.g., currents, sea surface temperature, sunlight, undisturbed water) as well as remote sensing data (e.g., bloom chlorophyll) and records of biotoxin monitoring programmes with health records to explore acute and chronic diseases possibly associated with HABs and human residence relative to coasts and other water bodies. The expansive scale in time and space, as well as the large sample sizes of the human health databases, could provide sufficient data to appropriately explore the climate change-HAB-human health hypothesis and other aspects (e.g., identification of potentially vulnerable populations, modelling for early HAB warnings, *etc.*) [52,53].

### 3.3. Potential uses for Public Health Professionals and Policymakers

Depending on the accessibility and types of data available, stakeholders for these data mashups include climate and health researchers, public health professionals, clinicians, policy makers, industry, and the military, and even the media, business, and the general public, depending on the interface and the mashup. In the future, these data linkages could lead to the availability of improved near term predictive models and better long term projections of health impacts of climate change, as well as the identification of geographic hotspots, for better prevention. In turn, this would enable forward planning of environmental and health resources in risk areas and populations, as well as the support of adaptive capacities to increase resilience of individuals, households, communities, and regions to the health effects of climate change.

The lack of linked data has prevented the identification of key relationships and limited the potential for early warning and planning, as well as the application and evaluation of potential interventions. In particular, as noted above, the data linkages may be able to demonstrate the potential benefits of the continuous and real time linkage of climate, environmental and health databases to perform active surveillance with active decision making, as well as the ability to explore a variety of hypotheses and interventions cost effectively and in quick succession.

## 4. Conclusions

To understand, forecast and adapt rapidly to climate, weather, and environmental events including impacts on the environment and on human health and wellbeing, new and evolving data mashups are needed to provide both the evidence base and decision support tools. The ethical, logistical and methodological challenges will need to be addressed continuously, as well as the architecture of data systems that could make them usable by a wide range of stakeholders (Table 2). There is thus the need for algorithms to enable genuine research questions to be investigated, with due consideration given for the confidentiality of individuals, access, ethics, or governance [54]. In particular, major aspects of data access, ownership and control (especially with regards to individual and subgroup confidentiality) need to be clarified. In order to ensure long term stability, it will be essential to clarify who pays for these data mashup resources, especially in the future. For example, there have been cases in which one publically-funded organization was instructed to make its data freely available because the data were generated using taxpayers money, while another publically-funded body of similar standing was instructed to charge for access to its data to ensure that the taxpayer received a return on their investment; in addition, there are ongoing costs to the access, updating, infrastructure, and storage of data in data mashups which must be covered by developing sustainable income streams by providing services and opportunities for research and training. It is important to clarify who has responsibility for developing and sustaining the appropriately trained personnel, together with the hardware and software. Processes for prioritizing the research questions, interpreting the findings, and implementing these findings where appropriate will need to be defined. 

**Table 2:** Potential challenges for and with data mashups.

Potential Challenges
*Creating and Maintaining the Mashup*
Mashup access, governance, and ownership
Access to and ownership of original data
Training of personnel and users
Rapidly changing hardware and software
Funding and resources (including long term secure data storage and appropriate staffing) to ensure longevity
*Data Issues*
Confidentiality of data
International standardization of data
Different types of complex data with issues of variable granularity, time spans, “richness”, certainty, *etc.*
Creation and maintenance of data documentation
Understanding of the uncertainty of the data
*Using the Mashup*
Need for and understanding of new methods of modeling and statistics
Interpretation of data, analyses and findings
Interpretation and evaluation of new associations for validity and strength
Use of real time data to make decisions
Evaluation of use and effectiveness of the mashup
Ability to look at big picture without obscuring smaller issues (such as effects on subpopulations)
Communication of the uncertainty of data and findings
Interactions with wide variety of stakeholders
Maintenance of the mashup and its resources over long periods of time

In the digital era, there is growing concern that potentially identifiable information is increasingly available without an individual’s consent. Real concerns center around these data mashups which are combinations of multiple data sources independent of each other, but which together could potentially reveal more as a whole than the sum of the individual parts. With smart-phone technology increasingly widely used, so called “Big Data” are becoming widely available at our finger-tips. There is now the potential to electronically track in space and time a user either covertly or overtly, for example when users manually enable geo-tagging in Twitter.

Researchers—and those elected to govern us—are able to deploy the massive amounts of data now available; some of it now citizen-generated as part of the “Big Data” social-media revolution [23,55,56]. Better understanding is needed not just about their creation, but also their manipulation, and how they should—and, perhaps even more importantly, should not—be used and interpreted, especially given their use (and frequent mis-use) in forecasts on which so many private and public sector plans are based [57].

**Table 3:** Summary table of health databases the MED MI Partnership has identified, as well as other databases for potential future collaborations (more details at http://bit.ly/OZwgxo).

Health	CPRD	UK Biobank	ONS Mortality	Million Women	DSSS	RCGP WRS	LABBASE PHE	ELS PHE	Vec S PHE
From	2012	2010	1836	1996	1999	1967	1975 *	1980	1900 **
Cohort	20M patients registered with general practitioners (projected)	503,316	n/a	1.36 m	n/a	n/a	n/a	n/a	n/a
Area	England	UK	England + Wales	UK	England + Wales	England + Wales	England	England	England
Info.	Underpins a comprehensive interventional research service. Extremely comprehensive and vital to this kind of linking research.	Age 40–69 Very broad range of genetic variables, phenotypic and exposure data.	All causes of death	Women 50–64. Special focus on HRT and breast health.	Self-reported, including cold, flu, fever, rash, heat *etc*. Provides early warning of infectious diseases	GP-diagnosis Gold standard of sentinel GP networks	Lab diagnosis Very large dataset, including known seasonal diseases e.g., giardia	Clinical diagnosis Additional demography and other context	Vector distr. In England Species host and number
Geo-ref	Postcode	Postcode	Postcode	Postcode	SHA ***	SHA ***	Postcode	Postcode	Grid Ref

DSSS: Direct Syndromic Surveillance System (Public Health England (PHE)/NHS Direct); PHE has access for surveillance, use for research to be negotiated with NHS Direct); RCGP WRS: Royal College of General Practitioners Weekly Returns Service; LABBASE: Laboratory confirmed diagnoses; ELS: Enhanced Legionella Surveillance; Vec S: Vectors Surveillance (ticks and mosquitoes); * Giardia (1975), Campylobacter (1976), Legionella (1977), Salmonella (1980), Cryptosporidium (1983), Lyme disease (1986); ** More intensive surveillance from 2005; *** Possible to use first part of postcode.

**Table 4:** Summary table of potential health databases the MED MI Partnership has identified, as well as other databases for potential future collaborations (more details at http://bit.ly/OZwgxo).

Health (Possible Future Collaborations)	ARS	1958 Birth Cohort	ALSPAC	ELSA	CFAS I, II	White-Hall II
From	2000	1958	1991	2002	1989	1985
Cohort	n/a	17,416	14,000	13,500	18 k	10,308
Area	UK	GB	Avon		UK	UK/London
Information	Response times to 999 calls (weather-related)	Single week, with follow-ups	Strong environmental and genetic data	Age > 50. Health and social. Ongoing study with new recruits.	Age 65+. Genetic and other data. Focus on dementia	Age 35–55 in 1985–1988 Civil Service staff
Geo-ref	Postcode	Wards	Postcode	Postcode	Postcode	Postcode

ARS: Ambulance Response Data (12 regional trusts); ALSPAC: Avon Longitudinal Study Parents and Children; ELSA: English Longitudinal Study of Ageing; CFAS: Cognitive Function and Ageing Studies.

**Table 5:** Summary table of environmental databases the MED MI Partnership has identified, as well as other databases for potential future collaborations (more details at http://bit.ly/OZwgxo).

Environment	MIDAS (Fixed Station Observations)	Pollen (Station Observations)	Daily Land Gridded 5 km	Monthly Land Gridded 5 km	Daily Sea Surface Temperature Gridded 5 km	Marine Biotoxins (Station Observations)
From	1961 (a few further back to 1850)	<1950	1961	1961	1985	2001: England & Wales; 2005: Scotland
Area	UK and coastal ships	UK land	UK land	UK land	Global Ocean	UK coastal locations
Availability	Research License via BADC or from Met Office	Owned by MAARA/Pollen UK (see letter of support)	Research License from Met Office	Research License from Met Office	Freely available through MyOcean.	Owned by CEFAS on behalf of Food Standards Authority
Information	450 stations supply daily: mean, maximum & minimum temperatures; sunshine amount; snow depth at 09:00 UTC 250 UK stations supply hourly: temperature; wind; cloud base & cover; visibility; weather type 10 marine stations supply sea surface temperature 3,000 UK stations supply daily precipitation data Boundary layer stability (for pollution dispersion) can be estimated for 250 UK stations. Daily shortwave radiation and daily erythemic UV radiation can be estimated for 450 UK stations.	Over three decades of data on airborne pollen and fungal spores. Longest running aerobiology datasets with strong links to Leicester Institute for Lung Health. Derby/Leicester data available for free; other data series from London and Wales may be negotiable.	Daily mean temperature, daily max temperature, daily minimum temperature, precipitation amount all provided for each 5 km grid square. Temperature data available free. License charge for precipitation data.	Precipitation amount, weather type, sunshine amount, provided for each month for each 5 km grid square. Daily shortwave radiation and daily erythemic UV radiation can be estimated.	Sea Surface Temperature retrieved from a combination of remote and in situ measurement at a resolution of ^1^/_20_ degree (~5 km).	Sampling records for a variety of sites around the UK coastline. Changes in sampling practice make year-to-year trends difficult to extract, but case study comparison with simulated results should be possible. Access to data by negotiation with CEFAS. Homogenization of the series will require resources beyond the scope of MED MI.
Geo-ref	Latitude & longitude; height above mean sea level	Latitude & longitude; height above mean sea level			Latitude & Longitude	Latitude & Longitude

Opportunities and concerns such as these have led to a number of initiatives to address the challenges and opportunities, including in the UK. Notably the UK Administrative Data Task Force suggested in their 2012 report that Administrative Data Research Centres (ADRC) should be established in each of the four countries in the UK with responsibility “*for commissioning and undertaking linkage of data from different government departments, and making the linked data available for analysis, thereby creating new resources for a growing research agenda*” (http://www.esrc.ac.uk/_images/ADT-Improving-Access-for-Research-and-Policy_tcm8-24462.pdf).

Based on the “history” of data mashups to date, both unintended consequences and new uses will emerge with both positive and negative ramifications. Nevertheless, the breadth and complexity of climate change and health issues require a new approach to the evidence base and decision support tools. MED MI and other initiatives to exploit the potential for data linkage can usher in an era of improved understanding of the impacts of climate change and facilitate attempts to adapt as far as possible.
